# General Result of the Vaccinations and Re-vaccinations in France

**Published:** 1841-08

**Authors:** 


*General result of the Vaccinations and Re-vaccinations in France.*—There is in a recent number of the *Annales d'Hygiene et de Med. Legale* a table prepared by M. VILLENEUVE, the reporter of a commission appointed by the Royal Academy of Medicine, exhibiting the general results of the vaccinations and re-vaccinations performed, and of the number and issue of the cases of small pox in those who had been vaccinated, compiled from reports sent from forty-one departments. The results of those reports only are given in which the vaccinators have recorded their unsuccessful as well as successful cases; and wherever the re-vaccinations were described as doubtful, they have been omitted.

We give only the totals.

| Vaccinations. | | | Re-vaccinations after ascertained Vaccination. | | | Smallpox after ascertained Vaccination. | |
|---|---|---|---|---|---|---|---|
| Number. | Successful. | Unsuccessful. | Number. | Successful. | Unsuccessful. | Number. | Deaths. |
| 30,410 | 28,853 | 560 | 2199 | 223 | 1976 | 365 | 8 |

It results from this table:—

1. That the proportion of cases in which vaccination failed, compared with that in which it took effect—estimated by some writers as one to eight, or one to ten—is not more than about one to fifty-four.

2. That of 2199 cases, in which re-vaccination was performed on persons of different ages and sexes who had been successfully vaccinated at some previous period of their lives, the operation took effect in 223 cases only—which would give the proportion of about one to thirteen or fourteen.

3. That of 365 cases of confirmed small pox, occurring in persons who had been at some previous period successfully vaccinated, there were only eight that proved fatal—giving a proportion of about one in forty-five or forty-six

We know that sporadic small pox usually carries off about an eigth or a tenth of those who are affected with it; and that, when the disease becomes epidemic, the mortality is often as high as one in four, and sometimes even higher.

M. Villeneuve, in submitting the above table as containing the results of the labours of the commission, admits that the data hitherto supplied are far from being sufficient to solve the question submitted by the government to the Royal Academy—whether it is necessary to have recourse to re-vaccination as a universal measure throughout France.—*Am. Journ. Med. Sciences.*

